# A Novel Synthesis of Fused Uracils: Indenopyrimidopyridazines, Pyrimidopyridazines, and Pyrazolopyrimidines for Antimicrobial and Antitumor Evalution

**DOI:** 10.3390/molecules21121714

**Published:** 2016-12-14

**Authors:** Samar El-Kalyoubi, Fatimah Agili

**Affiliations:** 1 Department of Organic Chemistry, Faculty of Pharmacy (Girls), Al-Azhar University, Nasr City 11651, Cairo, Egypt; 2 Medical Chemistry Department, Faculty of Medicine (Female Section), Jazan University, Jazan 45142, Saudi Arabia; 3 Chemistry Department, Faculty of Science (Female Section), Jazan University, Jazan 82621, Saudi Arabia; dr.fatmah2000@gmail.com

**Keywords:** 6-chlorouracil, 6-hydrazinyluracils, ninhydrin, isatin indenopyrimidopyridazines, pyrrolopyrimidines, pyrimidopyridazines

## Abstract

A variety of different compounds of fused uracils were prepared simply by the heating of 6-hydrazinyl-1-methyl-, 6-hydrazinyl-1-propyl-, or 6-hydrazinyl-1,3-dipropyluracil under reflux with ninhydrin, isatin, benzylidene malononitrile, benzylylidene ethyl cyanoacetate, benzil, and phenacyl bromide derivatives. The newly synthesized compounds were completely screened for antimicrobial and antitumor activity.

## 1. Introduction

For the last several decades, fused pyrimidine derivatives have become a significant attraction in the field of medicinal chemistry research. This is attributed to the fact that pyrimidine is the basic unit of DNA and RNA structure. This fact explains the wide range of pharmacological activities of pyrimidine derivatives. Pyrazolo[3,4-*d*]pyrimidine derivatives are a class of fused pyrimidines possessing significant biological activities [1,2]. They act as purine analogs [3] and many of their derivatives act with antimicrobial [2,4,5], antiviral [1,6], antimetabolites [7], anticancer [8,9], anti-inflammatory [10,11,12], and xanthine oxidase inhibitor activities [13,14].

Furthermore, pyrimidopyridazine derivatives have a significant interest owing to the fact that they have a potent pharmacological effect as therapeutic agents [15,16,17]. They have monoamine oxidase (MAO) inhibitory effect and subsequent modification on the diazine ring results in different inhibitory activities [18]. MAO inhibitory drugs play an important role in clinical management of depression, as well as Alzheimer’s disease [19].

It has been established that cancer is spread worldwide and responsible for about 15% of all deaths [20]. Many drugs with anticancer and antiviral activities have been developed [21], such as zidovudine (AZT) [22], zalcitabine (DDC) [23], brivudine (BVDU) [24], and methotrexate (MTX) [25]. 4-Deazatoxaflavin (1,6-dimethyl-1,5,6,7-tetrahydropyrimido[4,5-*c*]pyridazine-5,7-dione) binds to herring sperm DNA and inhibits growth of *Pseudomonas* 568 [21,26].

On account of these facts, a new series of substituted pyrimidopyridazines and pyrazolopyrimidines have been synthesized starting from 6-hydrazinyluracil derivatives and their antimicrobial, as well as antitumor activity has been evaluated and reported.

## 2. Results and Discussion

### 2.1. Chemistry

**Scheme 1:**
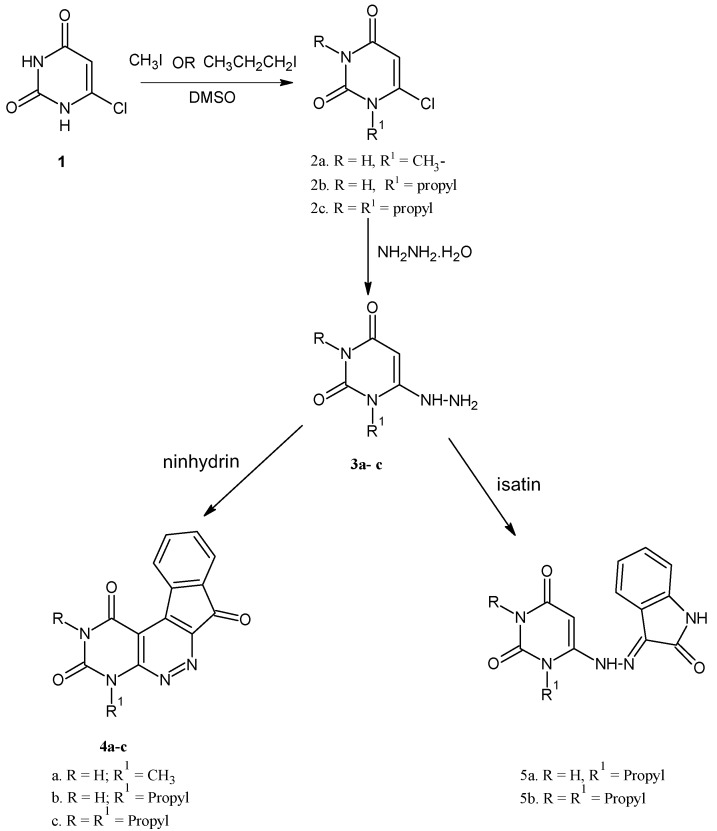
The reaction of 6-hydrazinyluracils with ninhydrin and isatin.

Extending our work in the synthesis of non-nucleosidic compounds of fused uracils [27,28,29], we tried to synthesize pyrimidopyridazines and pyrazolopyrimidines from 6-hydrazinyluracils. The regioselective alkylation of 6-chlorouracil **1** [30] with methyl—and/or propyl iodide in dimethyl sulfoxide (DMSO) in the presence of K_2_CO_3_ as basic medium afforded about 60%–70% yield of alkylated uracils **2a**–**c** [31,32,33,34]. The nucleophilic substitution of 6-chlorouracil **2a**–**c** using hydrazine hydrate afforded 6-hydrazinyluracils **3a**–**c** [34,35]. Heating of **3a**–**c** under reflux with ninhydrin for 5–10 min in the presence of AcOH resulted the desired compounds **4a**–**c** in good yield, as shown in Scheme 1. The structure of which was confirmed on the basis of analytical and spectral data. Thus, the ^1^H-NMR (DMSO-*d*_6_) spectrum of compounds **4a**,**b** showed a singlet around δ 12.24–12.23 ppm exchangeable characteristic for NH, while in **4c** a triplet splitting signal at δ 4.36 ppm characteristic for -NCH_2_ of propyl group. Additionally, a characteristic signal of the phenyl group for compounds **4a**–**c** appears around δ 9.26–7.75 ppm and the characteristic signal disappeared at 5–6 ppm of CH (5) in compounds **4a**–**c**. ^13^C-NMR showed 14 signals for compound **4a** and 16 signals for **4b** characteristic for carbon atoms. While, refluxing of **3b**,**c** with isatin for 1–2 h in AcOH gave the open form **5a**,**b** as shown in Scheme 1. Compounds **5a**,**b** were proved by the ^1^H-NMR spectrum, which showed three singlets at δ 13.01, 11.35, 10.99 ppm characterized for three NH groups, a singlet at δ 5.61 characterized for CH(5) of compound **5a** and two singlets at δ 13.03, 11.37 ppm characteristic for two NH groups, a singlet at δ 5.74, characterized for CH(5) of compound **5b**. ^13^C-NMR showed 15 signals for compound **5a** and 18 signals for **5b** characteristic for carbon atoms.

**Scheme 2:**
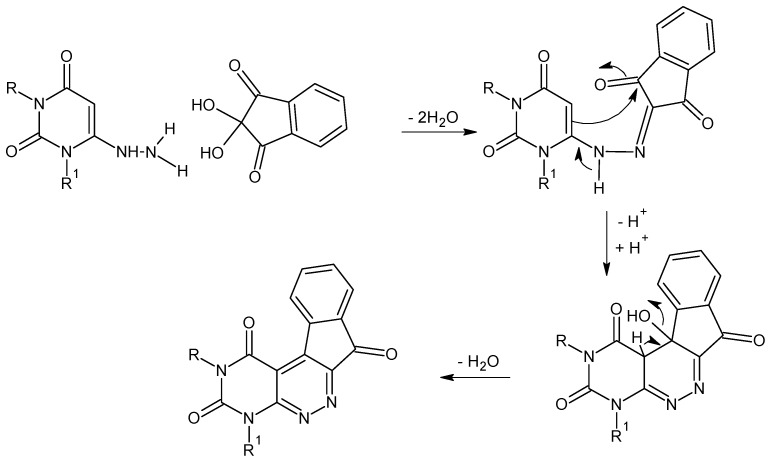
The mechanism formation of **4a**–**c**.

The mechanism formation of **4a**–**c** is shown in Scheme 2.

**Scheme 3:**
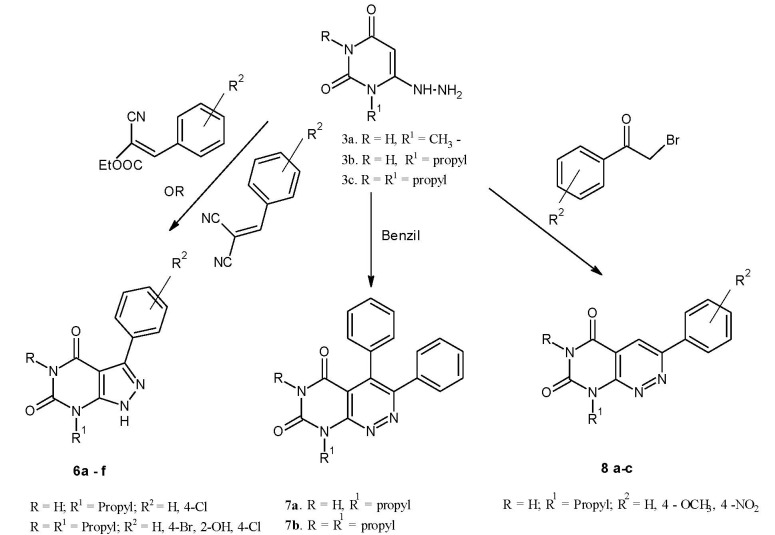
The reaction of 6-hydrazinyluracil with benzylidene malononitrile, benzylidene ethyl cyanoacetate, benzil, and phenacyl bromides.

**Scheme 4:**
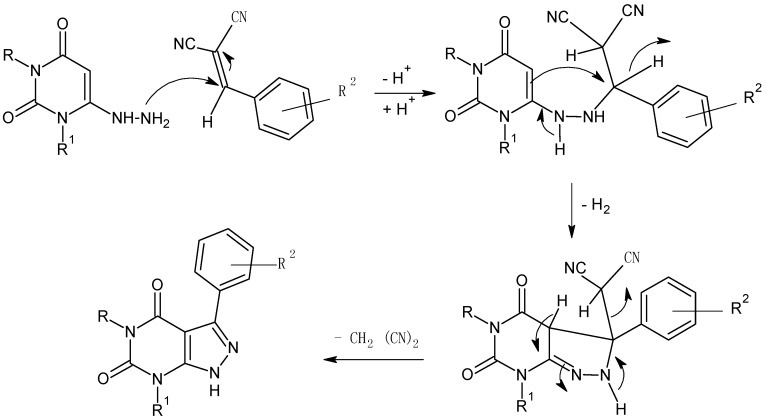
The expected mechanism for the reaction of 6-hydrazinyluracil with malononitrile.

On the other hand, the reaction of **3b**,**c** with even benzylidene malononitrile or benzylidene ethyl cyanoacetate derivative via Michael addition reaction by heating under reflux for 6–8 h in dimethylformamide (DMF) in the presence of triethylamine as basic medium furnished the same products of pyrazolopyrimidines **6a**–**f**, as shown in Scheme 3 by the elimination of malononitrile and ethyl cyanoacetate moieties, respectively, as shown in Scheme 4. Compounds **6a**–**f** were confirmed on the basis of analytical and spectral data. The ^1^H-NMR spectrum showed a characteristic singlet at δ 10.95, 11.19 ppm for NH(5) of compounds **6a** and **6b**, respectively, a singlet around δ 7.95–7.50 ppm for NH(1) and characteristic signals for the phenyl group around δ 7.73–6.72 ppm for compounds **6a**–**f**. ^13^C-NMR for compounds **6b** and **6e** showed 12 and 17 signals characteristic for carbon atoms respectively.

Heating of **3b**,**c** under reflux conditions for 4–5 h with benzil in dimethylformamide in the presence of triethylamine furnished **7a**,**b** in moderate yield, as shown in (Scheme 3). On the other hand, compound **7a** was also obtained via heating of **3b** with α-phenyl phenacyl bromide under reflux conditions for 5 h. Compounds **7a**,**b** were confirmed on the basis of analytical and spectral data. ^1^H-NMR spectra of **7a** showed a singlet at δ 11.72 characteristic for NH(6) and signals of phenyl groups for **7a**,**b** around δ 7.26–7.10 ppm. ^13^C-NMR showed 16 signals characteristic for carbon atoms of compound **7a**.

Finally, reaction of **3b** with different phenacyl bromides such as phenacyl-, *p*-methoxyphenacyl-, and *p*-nitrophenacyl bromide by heating under reflux for 4–6 h in dimethylformamide in the presence of triethylamine afforded pyrimidopyridazines **8a**–**c** in moderate yields (Scheme 3). Compounds **8a**–**c** were identified on the basis of analytical and spectral data. The ^1^H-NMR spectra showed a singlet around δ 12.08–11.96 ppm characteristic for NH(6), a characteristic singlet aromatic proton at CH(4) around δ 8.69–8.43 ppm of pyridazine ring, and signals of phenyl groups around δ 8.69–7.00 ppm. ^13^C-NMR showed 14 signals for compound **8b** characteristic for carbon atoms.

The expected mechanism for the reaction of 6-hydrazinyl uracil with benzylidene malononitrile and/or benzylidene ethyl cyanoacetate (Scheme 4).

### 2.2. Antimicrobial Screening

As shown in Table 1, the newly-synthesized compounds tested displayed variable in vitro antibacterial and antifungal activities. From the screening results, it can be seen that compound **4b** showed the highest activity against Gram-positive bacteria *Bacillus subtilis* compared with the standard drug, followed by compounds **4c**, **5a**, **6b**, **6d**, **4a**, **5b**, **6c** and **6f**, respectively. Similarly, compound **4b** showed the highest activity against Gram-positive bacteria *Streptococcus pneumonia* in comparison to the standard drug, followed by compounds **5a**, **4c**, **6b**, **6d**, **6c**, **4a**, and **5b**, respectively. On the other hand, compound **4b** showed the highest activity against Gram-negative bacteria *Escherichia coli* compared with the standard drug, followed by compounds **4c**, **6b**, **5a**, **6d**, **4a**, and **6c**, respectively. However, the order of activity against *Pseudomona aeruginosa* was **4b**, followed by compounds **5a**, **4c**, **6b**, **6d**, **6c**, **4a**, and **5b**, respectively. Regarding the activity of the tested compounds against the tested filamentous fungus *Aspergillus fumigatus*, the order of activity being **4b**, **4c**, **6b**, **5a**, **6d**, **4a**, **6c**, **6f**, respectively. Compound **6f** showed a weak antimicrobial effect on Gram-positive bacteria *Bacillus subtilis* as well as the tested filamentous fungus *Aspergillus fumigatus*. No antimicrobial activities were detected for compounds **7a** and **7b**. None of the tested compounds exert any activity against the pathogenic yeast species (*Candida albicans*) under these screening conditions.

The minimum inhibitory concentration of the six most active synthesized compounds were detected, as shown in Table 2. It was shown that **4b** showed the highest potential where its minimum inhibitory concentration (MIC) was comparable with that of the standard compounds, whereas **4a** showed the lowest potential and a very high MICs in comparison to the standard.

#### Anticancer Activity

**Figure 1:**
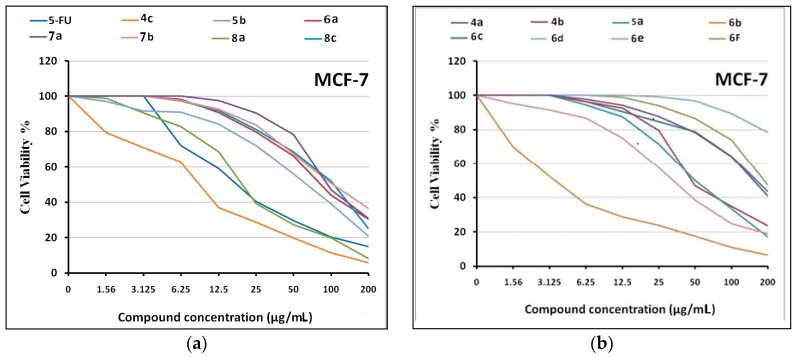
(**a**) The dose response curve showing the in vitro inhibitory activity of the tested compounds **4c**, **5b**, **6a**, **7a**, **7b**, **8a** and **8c** against breast carcinoma (MCF-7) cell line compared with the reference drug 5-flourouracil; (**b**) The dose response curve showing the in vitro inhibitory activity of the tested compounds **4a**, **4b**, **5a** and **6b**–**f** against breast carcinoma (MCF-7) cell line compared with the reference drug 5-flourouracil.

The in vitro growth inhibitory activity of the synthesized compounds was investigated in comparison with the well-known anticancer standard drug 5-flourouracil under the same conditions using colorimetric viability assay. Data generated were used to plot a dose response curve of which the concentration of test compounds required to kill 50% of cell population (IC_50_) was determined. The results revealed that all the tested compounds showed inhibitory activity to the tumor cell lines in a concentration dependent manner. Cytotoxic activity was expressed as the mean IC_50_ of three independent experiments. The results are represented in Table 3 and Figure 1a,b showed that compound **4a** was the most active against the breast carcinoma cell line (MCF-7), compared with the reference drug with IC_50_ values of 3.6 and 4.1 μg/mL, respectively. Interestingly, compounds **4a**, **4c**, and **8a** exhibited potent antitumor activity against breast cancer, respectively, and were the most active among their analogues. Moreover, the other compounds were less active.

## 3. Experimental Section

### 3.1. General

All melting points were determined with an Electrothermal Mel.-Temp. II (Registered trademark of Barnstead, Barnstead, NH, USA) apparatus and were uncorrected. Element analyses were performed at Regional Center for Mycology and Biotechnology at Al-Azhar University. The infrared (IR) spectra were recorded using a potassium bromide disc technique on a Nikolet IR 200 FT IR spectrometer (Thermo Electron Scientific Instruments LLC, Madison, WI, USA) and carried out in Taif University, Taif, KSA. Mass spectra were recorded on DI-50 unit of Shimadzu GC/MS-QP 5050A mass spectrometer (Shimadzu Corporation, Tokyo, Japan) at the Regional Center for Mycology and Biotechnology at Al-Azhar University. ^1^H-NMR and ^13^C-NMR spectra were recorded in DMSO-*d*_6_ as a solvent using a Varian Mercury spectrometer at 400 MHz and 125 MHz, respectively, Applied Nucleic Acid Research Center, Zagazig University, Egypt. Chemical shifts (δ) are given in ppm and coupling constants (*J*) are given in Hz. All reactions were monitored by TLC using pre-coated plastic sheet silica gel (0.25 mm, 20 × 20 cm, 60F_254_, E. Merck KGaA, Konstanz, Germany) and spots were visualized by irradiation with UV light (254 nm). The used solvent system was chloroform:methanol (9:1) and ethyl acetate:toluene (1:1).

6-Chlorouracil (**1**) was prepared according to the reported method [30].

*6-Chloro-1-alkyl- and/or 1,3-Dialkyluracils* **2a**–**c** [31,32,33,34]

*6-Chloro-1-propyluracil* (**2b**) and *6-chloro-1,3-dipropyluracil* (**2c**): A solution of 6-chlorouracil (**1**) (40 mmol) in dimethyl sulfoxide (25 mL) was heated gently until 6-chlorouracil dissolved, and then potassium carbonate (20 mmol) was added with stirring. Propyl iodide (40 mmol) was added one time and the mixture was stirred at room temperature for 6 h. Water (40 mL) was added, and cooled in an ice box for several hours. The formed precipitate was collected by filtration, washed with water, dried in the oven at 80 °C, and crystallized from methanol to give 3.8 g of a white crystalline precipitate (51% yield) **2b** with m.p. = 165 °C.

The mother liquor was evaporated in vacuo until dryness, then water (30 mL) was added, followed by extraction with chloroform (40 mL × 3). The chloroformic layer was evaporated and the obtained colorless crystals was dried in desiccator to give 2.3g (25%) of **2c** with m.p. = 58 °C; ^1^H-NMR (DMSO-*d*_6_) δ ppm: 6.03 (s, 1H, CH-5), 4.26 (t, 2H, NCH_2_), 3.89 (t, 2H, NCH_2_), 1.52–1.62 (m, 4H, 2CH_2_), 0.84–0.88 (m, 6H, 2CH_3_).

*6-Hydrazinyl-1-methyl-, 1-Propyl- and/or 1,3-Dipropyluracils* (**3a**–**c**) [34,35]

**3a**: Yield 95%; m.p. 254 °C, lit. [34] = 255 °C; **3b**: Yield 84%; m.p. 238–240 °C; **3c**: Yield 91%; m.p. 120 °C.

*2,4-Disubstituted-1H-indeno[2,1-c]pyrimido[5,4-e]pyridazine-1,3,7(2H,4H)-triones* (**4a**–**c**)

A mixture of 6-hydrazinyl-1-substituted and/or -1,3-disubstituteduracils (**3a**–**c**) (1.9 mmol) and ninhydrin (1.9 mmol) in acetic acid (5 mL) was heated under reflux for 5–10 min. The formed precipitate after cooling was filtered, washed with ethanol and crystallized from DMF/ethanol (1:3).

*4-Methyl-1H-indeno[2,1-c]pyrimido[5,4-e]pyridazine-1,3,7(2H,4H)-trione* (**4a**): Yield: 48%; m.p. >300 °C; IR (KBr) ν_max_ (cm^−1^): 3178 (NH), 3062 (CH arom), 2885 (CH aliph), 1687, 1631, 1597 (C=O), 1447 (C=C); ^1^H-NMR (DMSO-*d*_6_) δ ppm: 12.24 (s, 1H, NH),9.20 (d, 1H, *J* = 7.6 Hz), 7.89–7.76 (m, 3H, arom), 3.66 (s, 3H, CH_3_); ^13^C-NMR (DMSO-*d*_6_): δ = 188.2, 160.9, 153.5, 151.2, 149.8, 140.8, 137.5, 136.5, 134.8, 133.9, 131.4, 129.2, 109.9, 29.9; MS: *m*/*z* (%) = M^+^, 280 (52), 251 (10), 182 (59), 155 (52), 154 (28), 153 (22), 138 (100), 126 (32), 111 (23), 99 (20), 76 (37). Anal. Calcd for C_14_H_8_N_4_O_3_: C, 60.00; H, 2.88; N, 19.99. Found: C, 60.16; H, 2.85; N, 20.14.

*4-Propyl-1H-indeno[2,1-c]pyrimido[5,4-e]pyridazine-1,3,7(2H,4H)-trione* (**4b**): Yield: 51%; m.p. 281–283 °C; IR (KBr) ν_max_ (cm^−1^):3174 (NH), 3047 (CH arom), 2974, 2838 (CH aliph), 1720, 1692, 1555 (C=O), 1458 (C=C); ^1^H-NMR (DMSO-*d*_6_) δ ppm: 12.23 (s, 1H, NH, exchangeable), 9.23 (d, 1H, *J* = 7.6 Hz), 7.90–7.75 (m, 3H, arom), 4.31 (t, 2H, *J* = 7.6 Hz, CH_2_), 1.77–1.71 (m, 2H, *J* = 7.6 Hz, CH_2_), 0.96 (t, 3H, 7.6 Hz, CH_3_). ^13^C-NMR (DMSO-*d*_6_): δ =188.1, 160.8, 153.5, 151.1, 149.6, 141.0, 137.6, 136.5, 134.7, 133.9, 131.4, 129.2, 110.0, 44.0, 20.4, 11.1; MS: *m*/*z* (%) = M^+^, 308 (100), 267 (94), 266 (80), 265 (36), 238 (50), 223 (77), 210 (32), 196 (48), 195 (34), 181 (49), 167 (36), 155 (33), 154 (37), 153 (15), 152 (24), 139 (49), 138 (39), 127 (36), 126 (53), 125 (46), 112 (24), 99 (28). Anal. Calcd for C_16_H_12_N_4_O_3_: C, 62.33; H, 3.92; N, 18.17. Found: C, 62.51; H, 3.95; N, 18.25.

*2,4-Dipropyl-1H-indeno[2,1-c]pyrimido[5,4-e]pyridazine-1,3,7(2H,4H)-trione* (**4c**): Yield: 71%, m.p. 260–262 °C; IR (KBr) ν_max_ (cm^−1^): 3050 (CH arom), 2961, 2873 (CH aliph), 1710, 1667, 1566 (C=O), 1432 (C=C); ^1^H-NMR (DMSO-*d*_6_) δ ppm: 9.26 (d, 1H, *J* = 7.6 Hz, arom), 8.11–7.61 (m, 3H, arom), 4.37 (t, 2H, *J* = 6.8 Hz, CH_2_), 3.95 (t, 2H, *J* = 6.8 Hz, CH_2_), 1.76–1.73 (m, 2H, CH_2_), 1.66–1.65 (m, 2H, CH_2_), 0.96 (t, 3H, *J* = 6.8 Hz, CH_3_), 0.93 (t, 3H, *J* = 6.8 Hz, CH_3_); MS: *m*/*z* (%) = M^+^, 350 (62), 323 (33), 290 (27), 268 (28), 262 (50), 240 (20), 238 (16), 236 (42), 223 (26), 209 (17), 196 (15), 195 (19), 192 (47), 180 (63), 177 (41), 169 (64), 154 (21), 140 (22), 138 (83), 123 (46), 112 (45), 99 (17), 180 (63), 98 (45), 97 (47), 94 (53), 74 (81), 73 (100); Anal. Calcd for C_19_H_18_N_4_O_3_: C, 65.13; H, 5.18; N, 15.99. Found: C, 65.45; H, 5.24; N, 16.17.

*6-(2-(2-Oxoindolin-3-ylidene)hydrazinyl)-1-propyl- and/or 1,3-Dipropylpyrimidine-2,4(1H,3H)-diones* **5a**,**b**

A mixture of 6-hydrazinyl-1-propyl- and/or 1,3-dipropyluracils (**3b**,**c**) (1.6 mmol) and isatin (1.6 mmol) in acetic acid (5 mL) was heated under reflux for 1–2 h. The formed precipitate after cooling was filtered, washed with ethanol, and crystallized from DMF/ethanol (1:3).

*6-(2-(2-Oxoindolin-3-ylidene)hydrazinyl)-1-propylpyrimidine-2,4(1H,3H)-dione* (**5a**): Yield: 53%; m.p. >300 °C; IR (KBr) ν_max_ (cm^−1^): 3195 (br., NH), 3095 (CH arom), 2956, 2815 (CH aliph), 1702, 1594, 1515 (C=O), 1458 (C=C); ^1^H-NMR (DMSO-*d*_6_) δ ppm: 13.01 (s, 1H, NH), 11.35 (s, 1H, NH), 10.99 (s,1H, NH), 7.63 (d, 1H, *J* = 7.6 Hz, arom), 7.39–7.36 (m, 1H, arom), 7.12–7.09 (m, 1H, arom), 6.97 (d, 1H, *J* = 7.6 Hz, arom), 5.61 (s, 1H, CH-5), 3.81 (t, 2H, *J* = 7.6 Hz, CH_2_), 1.69–1.64 (m, 2H, *J* = 7.6 Hz, CH_2_), 0.94 (t, 3H, *J* = 7.6 Hz, CH_3_); ^13^C-NMR (DMSO-*d*_6_): δ= 163.5, 162.3, 162.3, 151.0, 141.9, 136.1, 131.4, 122.7, 120.7, 119.5, 111.3, 78.5, 42.8, 20.9, 10.7; MS: *m*/*z* (%) = M^+^, 313 (21), 285 (34), 253 (30), 243 (16), 226 (11), 213 (12), 200 (14), 158 (10), 147 (14), 145 (19), 132 (13), 118 (39), 117 (35), 104 (34), 103 (22), 101 (19), 90 (32), 77 (47), 76 (29), 68 (100); Anal. Calcd for C_15_H_15_N_5_O_3_: C, 57.50; H, 4.83; N, 22.35. Found: C, 57.78; H, 4.90; N, 22.52.

*6-(2-(2-Oxoindolin-3-ylidene)hydrazinyl)-1,3-dipropylpyrimidine-2,4(1H,3H)-dione* (**5b**): Yield: 65%, m.p. 283–285 °C; IR (KBr) ν_max_ (cm^−1^):3137 (br., NH), 3084 (CH arom), 2966, 2877 (CH aliph), 1691, 1600, 1542 (C=O), 1458 (C=C); ^1^H-NMR (DMSO-*d*_6_) δ ppm: 13.03 (s, 1H, NH), 11.37 (s, 1H, NH), 7.95–7.87 (m, 1H, arom), 7.65 (d, 1H, *J* = 6.8 Hz, arom), 7.40–7.36 (m, 1H, arom), 6.98 (d, 1H, *J* = 6.8 Hz, arom), 5.74 (s, 1H, CH-5), 4.25 (t, 2H, *J* = 7.4 Hz, NCH_2_), 3.87 (t, 2H, *J* = 7.4 Hz, NCH_2_), 1.76–1.69 (m, 2H, *J* = 7.4 Hz, CH_2_), 1.54–1.53 (m, 2H, *J* = 7.4 Hz, CH_2_), 0.96–0.89 (m, 6H, 2CH_3_); ^13^C-NMR (DMSO-*d*_6_): δ = 163.5, 161.2, 158.0, 149.6, 141.9, 136.2, 131.4, 122.7, 120.7, 119.4, 111.3, 78.1, 43.2, 41.8, 20.1, 19.6, 11.1, 10.7; MS: *m*/*z* (%) = M^+^, 355 (86), 338 (20), 327 (47), 313 (24), 285 (34), 243 (59), 227 (20), 226 (12), 213 (27), 200 (52), 187 (36), 186 (54), 172 (12), 166 (41), 161 (100), 158 (32), 153 (31), 148 (50), 147 (42), 146 (17), 145 (69), 125 (17), 118 (42), 117 (44), 111 (66); Anal. Calcd for C_18_H_21_N_5_O_3_: C, 60.83; H, 5.96; N, 19.71. Found: C, 61.07; H, 6.03; N, 19.94.

*3-Substituted-7-propyl- and/or 5,7-Dipropyl-1H-pyrazolo[3,4-d]pyrimidine-4,6(5H,7H)-diones* **6a**–**f**

Method A: A mixture of 6-hydrazinyl-1-propyl- and/or 1,3-dipropyluracils (**3b**,**c**) (1.7 mmol) and appropriate benzylidene malononitriles (1.7 mmol) in DMF (5 mL) in the presence of TEA (1mL) was heated under reflux for 6–8 h. The reaction mixture was evaporated under reduced pressure. The residue was treated with ethanol (10 mL), the formed precipitate was filtered, washed with ethanol, and crystallized from DMF/ethanol (2:1) to afford **6a**–**f**.

Method B: A mixture of 6-hydrazinyl-1,3-dipropyluracil (**3c**) (1.7 mmol) and 4-chlorobenzylidene ethyl cyanoacetate (1.7 mmol) in DMF (5 mL) in the presence of TEA (1 mL) was heated under reflux for 8 h. The reaction mixture was evaporated under reduced pressure. The residue was treated with ethanol (10 mL), the formed precipitate was filtered, washed with ethanol, and crystallized from DMF/ethanol (2:1) to afford **6f**.

*3-Phenyl-7-propyl-1H-pyrazolo[3,4-d]pyrimidine-4,6(5H,7H)-dione* (**6a**): method A: Yield: 72%; m.p. >300 °C; IR (KBr) ν_max_ (cm^−1^): 3170 (br., NH), 3058 (CH arom), 2965 (CH aliph), 1679, 1595 (C=O), 1452 (C=C); ^1^H-NMR (DMSO-*d*_6_) δ ppm: 10.95 (s, 1H, NH), 7.95 (s, 1H, NH), 7.41–7.25 (m, 5H, arom), 3.84 (t, 2H, *J* = 7.2 Hz, NCH_2_), 1.71–1.69 (m, 2H, *J* = 7.2 Hz, CH_2_), 0.99 (t, 3H, *J* = 7.2 Hz, CH_3_); MS: *m*/*z* (%) = M^+^, 270 (4), 231 (8), 184 (25), 176 (10), 165 (19), 139 (10), 130 (11), 111 (23), 109 (14), 107 (11), 98 (17), 96 (16), 95 (17), 83 (30), 81 (23), 71 (30), 69 (99), 67 (26), 55 (100), 44 (20), 43 (86), 41 (75); Anal. Calcd for C_14_H_14_N_4_O_2_: C, 62.21; H, 5.22; N, 20.73. Found: C, 62.48; H, 5.24; N, 21.04.

*3-(4-Chlorophenyl)-7-propyl-1H-pyrazolo[3,4-d]pyrimidine-4,6(5H,7H)-dione* (**6b**): Method A: Yield: 69%; m.p. >300 °C; IR (KBr) ν_max_ (cm^−1^): 3215 (br., NH), 3050 (CH arom), 2965, 2869(CH aliph), 1684, 1614 (C=O), 1435 (C=C), 810 (*p*-substituted); ^1^H-NMR (DMSO-*d*_6_) δ: 11.19 (s, 1H, NH), 7.82 (s, 1H, NH), 7.47 (d, 2H, *J* = 8.6 Hz, arom), 7.29 (d, 2H, *J* = 8.6 Hz, arom), 4.05 (t, 2H, *J* = 7.4 Hz, NCH_2_), 1.65–1.63 (m, 2H, *J* = 7.4 Hz, CH_2_), 0.91 (t, 3H, *J* = 7.4 Hz, CH_3_); ^13^C-NMR (DMSO-*d*_6_) δppm: 160.4, 154.6, 150.3, 135.9, 133.0, 129.4, 128.7, 115.3, 99.2, 42.7, 20.6, 11.1; MS: *m*/*z* (%) = 306 (M+2, 9), M^+^, 304 (25), 271 (30), 265 (42), 211 (20), 176 (27), 145 (21), 138 (21), 131 (38), 125 (34), 116 (34), 114 (23), 110 (100), 87 (64), 84 (86), 82 (34), 43 (84), 42 (48); Anal. Calcd for C_14_H_13_ClN_4_O_2_: C, 55.18; H, 4.30; N, 18.39. Found: C, 55.37; H, 4.36; N, 18.57.

*3-Phenyl-5,7-dipropyl-1H-pyrazolo[3,4-d]pyrimidine-4,6(5H,7H)-dione* (**6c**): Method A: Yield: 77%; m.p. >300 °C; IR (KBr) ν_max_ (cm^−1^): 3195 (br., NH), 3040 (CH arom), 2964 (CH aliph), 1605, 1598 (C=O), 1449 (C=C); ^1^H-NMR (DMSO-*d*_6_) δ ppm: 7.95 (s, 1H, NH), 7.56–7.15 (m, 5H, arom), 3.89 (t, 2H, NCH_2_), 3.84 (t, 2H, *J* = 8.8 Hz, NCH_2_), 1.70–1.68 (m, 2H, CH_2_), 1.60–1.58 (m, 2H, CH_2_), 0.91–0.89 (m, 6H, 2CH_3_); MS: *m*/*z* (%) = M^+^, 312 (21), 282(18), 255 (20), 247 (14), 194 (31), 180 (16), 163 (46), 126 (17), 125 (60), 121 (33), 105 (22), 97 (77), 83 (42), 81 (19), 80 (38), 69 (58), 57 (31), 56 (100), 43 (71); Anal. Calcd for C_17_H_20_N_4_O_2_: C, 65.37; H, 6.45; N, 17.94. Found: C, 65.48; H, 6.53; N, 18.09.

*3-(4-Bromophenyl)-5,7-dipropyl-1H-pyrazolo[3,4-d]pyrimidine-4,6(5H,7H)-dione* (**6d**): Method A: Yield: 78%; m.p. >300 °C; IR (KBr) ν_max_ (cm^−1^): 3135 (NH), 3023 (CH arom), 2966, 2837 (CH aliph), 1678, 1676 (C=O), 1496 (C=C); ^1^H-NMR (DMSO-*d*_6_) δ ppm: 7.84 (s, 1H, NH, exchangeable), 7.73–7.21 (m, 4H, arom), 4.0–3.64 (m, 4H, 2NCH_2_), 1.68–1.61 (m, 4H, 2CH_2_), 0.91–0.80 (m, 6H, 2CH_3_), MS: *m*/*z* (%) = M^+^ + 2, 393 (1), M^+^, 391 (3), 355 (10), 327 (19), 283 (13), 262 (17), 220 (17), 207 (14), 160 (15), 159 (13), 157 (16), 141 (13), 129 (12), 119 (16), 115 (12), 109 (34), 97 (46), 95 (34), 87 (17), 85 (24), 84 (70), 81 (100), 71 (33); Anal. Calcd for C_17_H_19_BrN_4_O_2_: C, 52.19; H, 4.89; N, 14.32. Found: C, 52.53; H, 4.91; N, 14.39.

*3-(2-Hydroxyphenyl)-5,7-dipropyl-1H-pyrazolo[3,4-d]pyrimidine-4,6(5H,7H)-dione* (**6e**): Method A: Yield: 72%; m.p. >300 °C; IR (KBr) ν_max_ (cm^−1^): 3435 (OH), 3180 (NH), 3055 (CH arom), 2965 (CH aliph), 1605, 1545 (C=O), 1485 (C=C), 750 (*o*-substituted phenyl); ^1^H-NMR (DMSO-*d*_6_) δ ppm: 9.41 (s, 1H, OH), 7.59–6.72 (m, 5H, 1NH & 4H arom), 3.80–3.66 (m, 4H, 2NCH_2_), 1.67–1.65 (m, 2H, CH_2_), 1.52–1.50 (m, 2H, CH_2_), 1.16 (t, 3H, *J* = 7.6 Hz, CH_3_), 0.91 (t, 3H, *J* = 7.6 Hz, CH_3_); ^13^C-NMR (DMSO-*d*_6_): δ = 162.5, 158.0, 152.0, 151.9, 134.4, 125.3, 124.9, 118.6, 116.4, 115.9, 97.3, 43.1, 42.8, 20.2, 20.0, 11.1, 11.0; MS: *m*/*z* (%) = M^+^, 328 (57), 296 (23), 278 (48), 266 (15), 223 (14), 179 (14), 165 (15), 140 (10), 136 (14), 129 (16), 127 (17), 125 (20), 116 (31), 115 (25), 113 (20), 111 (31), 109 (23), 107 (27), 97 (33), 81 (19), 77 (40), 69 (77), 67 (48), 59 (20), 56 (94), 43 (100); Anal. Calcd for C_17_H_20_N_4_O_3_: C, 62.18; H, 6.14; N, 17.06. Found: C, 62.44; H, 6.21; N, 17.23.

*3-(4-Chlorophenyl)-5,7-dipropyl-1H-pyrazolo[3,4-d]pyrimidine-4,6(5H,7H)-dione* (**6f**): Method A: Yield: 69%, method B: Yield: 61%; m.p. >300 °C; IR (KBr) ν_max_ (cm^−1^ ): 3187 (NH), 3053 (CH arom), 2965, 2870 (CH aliph), 1685, 1613 (C=O), 1497 (C=C), 814 (*p*-substituted); ^1^H-NMR (DMSO-*d*_6_) δ ppm: 7.95 (s, 1H, NH), 7.52 (d, 2H, *J* = 8.8 Hz, arom), 7.32 (d, 2H, *J* = 8.8 Hz, arom), 4.99–3.81 (m, 4H, 2NCH_2_), 1.72–1.55 (m, 4H, 2CH_2_), 0.88–0.86 (m, 6H, 2CH_3_); MS: *m*/*z* (%) = M^+^ + 2, 348 (3), M^+^, 346 (8), 341(21), 314(25), 311 (16), 280 (29), 269 (17), 266 (23), 247 (22), 245 (17), 238 (25), 226 (20), 207 (18), 206 (19), 203 (30), 184 (22), 154 (33), 146 (33), 145 (40), 127 (56), 125 (26), 123 (25), 119 (43), 89 (82), 82 (100), 73 (51), 67 (63), 66 (45), 40 (87); Anal. Calcd for C_17_H_19_ClN_4_O_2_: C, 58.87; H, 5.52; N, 16.15. Found: C, 59.05; H, 5.61; N, 16.23.

*3,4-Diphenyl-8-propyl- and/or 6,8-Dipropylpyrimido[4,5-c]pyridazine-5,7(6H,8H)-diones* (**7a**,**b**)

Method A: A mixture of 6-hydrazinyl-1-propyl and/or 1,3-dipropyluracils (**3b**,**c**) (1.6 mmol) and benzil (1.6 mmol) in DMF (5 mL) in the presence of TEA (1 mL) was heated under reflux for 4–5 h. The reaction mixture was evaporated under reduced pressure. The residue was treated with ethanol (10 mL), the formed precipitate was filtered, washed with ethanol, and crystallized from DMF/ethanol (2:1) to afford compounds **7a**,**b**.

Method B: A mixture of 6-hydrazinyl-1-propyluracil (**3b**) (1.6 mmol) and α-phenylphenacyl bromide (1.6 mmol) in DMF (5 mL) in the presence of TEA (1 mL) was heated under reflux for 5 h. The reaction mixture was evaporated under reduced pressure. The residue was treated with ethanol (10 mL), the formed precipitate was filtered, washed with ethanol, and crystallized from DMF/ethanol (2:1) to afford **7a**.

*3,4-Diphenyl-8-propylpyrimido[4,5-c]pyridazine-5,7(6H,8H)-dione* (**7a**): Yield: method A: 76%, method B: 68%; m.p. 226–228 °C; IR (KBr) ν_max_ (cm^−1^): 3159 (NH), 3003 (CH arom), 2966, 2835 (CH aliph), 1674, 1538 (C=O), 1496 (C=C); ^1^H-NMR (DMSO-*d*_6_) δ ppm: 11.72 (s, 1H, NH), 7.25–7.10 (m, 10H, arom), 4.34 (t, 2H, *J* = 7.6 Hz, CH_2_), 1.82–1.77 (m, 2H, *J* = 7.6 Hz, CH_2_),0.99 (t, 3H, *J* = 7.6 Hz, CH_3_); ^13^C-NMR (DMSO-*d*_6_): δ = 159.8, 157.5, 151.7, 149.8, 139.3, 136.5, 134.6, 129.6, 128.9, 128.0, 127.6, 127.3, 111.7, 43.1, 20.5, 11.1; MS: *m*/*z* (%) = M^+^, 358 (16), 317 (10), 316 (46), 315 (100), 255 (10), 189 (9), 171 (9), 128 (4), 77 (5); Anal. Calcd for C_21_H_18_N_4_O_2_: C, 70.38; H, 5.06; N, 15.63. Found: C, 70.49; H, 5.10; N, 15.84.

*3,4-Diphenyl-6,8-dipropylpyrimido[4,5-c]pyridazine-5,7(6H,8H)-dione* (**7b**): Yield: method A: 64%; m.p. 220–222 °C; IR (KBr) ν_max_ (cm^−1^): 3056 (CH arom), 2963, 2873 (CH aliph), 1718, 1670 (C=O), 1495 (C=C); ^1^H-NMR (DMSO-*d*_6_) δ ppm: 7.26–7.10 (m, 10H, arom), 4.42 (t, 2H, *J* = 7.4 Hz, NCH_2_), 3.75 (t, 2H, *J* = 7.4 Hz, NCH_2_), 1.85–1.79 (m, 2H, *J* = 7.4 Hz, CH_2_), 1.53–1.47 (m, 2H, *J* = 7.4 Hz, CH_2_), 1.00 (t, 3H, *J* = 7.4 Hz, CH_3_), 0.83 (t, 3H, *J* = 7.4 Hz, CH_3_); MS: *m*/*z* (%) = M^+^, 400 (6), 383 (10), 369 (7), 267 (22), 223 (17), 135 (12), 133 (26), 127 (11), 125 (11), 112 (20), 110 (17), 101 (18), 95 (26), 90 (23), 86 (24), 83 (25), 80 (24), 76 (41), 72 (31), 70 (31), 69 (29), 59 (43), 55 (42), 44 (100), 42 (81), 40 (62); Anal. Calcd. for C_24_H_24_N_4_O_2_: C, 71.98; H, 6.04; N, 13.93. Found: C, 72.21; H, 6.12; N, 14.12.

*3-Substituted-8-propylpyrimido[4,5-c]pyridazine-5,7(6H,8H)-diones* **8a**–**c**

A mixture of 6-hydrazinyl-1-propyluracil (**3b**) (1.6 mmol) and appropriate phenacyl bromides (1.6 mmol) in DMF (5 mL) in the presence of TEA (1 mL) was heated under reflux for 4–6 h. The reaction mixture was evaporated under reduced pressure. The residue was treated with ethanol (10 mL), the formed precipitate was filtered, washed with ethanol, and crystallized from DMF/ethanol (2:1) to afford **8a**–**c**.

*3-Phenyl-8-propylpyrimido[4,5-c]pyridazine-5,7(6H,8H)-dione* (**8a**): Yield: 56%; m.p. 262–264 °C; IR (KBr) ν_max_ (cm^−1^): 3178 (NH), 3039 (CH arom), 2965, 2866 (CH aliph), 1668, 1592 (C=O), 1496 (C=C); ^1^H-NMR (DMSO-*d*_6_) δ ppm: 11.99 (s, 1H, NH), 8.50 (s, 1H, arom), 8.22–8.19 (m, 2H, arom), 7.57–7.46 (m, 3H, arom), 4.29 (t, 2H, *J* = 7.4 Hz, NCH_2_), 1.79–1.73 (m, 2H, *J* = 7.4 Hz, CH_2_), 0.97 (t, 3H, *J* = 7.4 Hz, CH_3_); MS: *m*/*z* (%) = M^+^, 282 (13), 254 (9), 241 (23), 240 (89), 239 (19), 197 (36), 77 (14), 44 (30), 40 (100); Anal. Calcd. for C_15_H_14_N_4_O_2_: C, 63.82; H, 5.00; N, 19.85. Found: C, 63.97; H, 5.08; N, 20.02.

*3-(4-Methoxyphenyl)-8-propylpyrimido[4,5-c]pyridazine-5,7(6H,8H)-dione* (**8b**): Yield; 61%; m.p. 219–221 °C; IR (KBr) ν_max_ (cm^−1^): 3162 (NH), 3039 (CH arom), 2967, 2826 (CH aliph), 1670, 1600 (C=O), 1449 (C=C), 838 (*p*-substituted); ^1^H-NMR (DMSO-*d*_6_) δ ppm: 11.96 (s, 1H, NH), 8.43 (s, 1H, arom), 8.17 (d, 1H, *J* = 7.0 Hz, arom), 7.46 (d, 1H, *J* = 8.4 Hz, arom), 7.10 (d, 1H, *J* = 7.0 Hz, arom), 7.01 (d, 1H, *J* = 8.4 Hz, arom), 4.28 (t, 2H, *J* = 7.4 Hz, NCH_2_), 3.84 (s, 3H, CH_3_), 1.78–1.72 (m, 2H, *J* = 7.4 Hz, CH_2_), 0.96 (t, 3H, *J* = 7.4 Hz, CH_3_); ^13^C-NMR (DMSO-*d*_6_): δ = 160.8, 160.1, 160.0, 150.9, 149.8, 131.1, 127.4, 126.3, 120.7, 114.5, 55.3, 42.8, 20.5, 11.1; MS: *m*/*z* (%) = M^+^, 312 (19), 271 (12), 270 (50), 269 (100), 255 (21), 239 (24), 135 (20), 40 (23); Anal. Calcd for C_16_H_16_N_4_O_3_: C, 61.53; H, 5.16; N, 17.94. Found: C, 61.71; H, 5.23; N, 18.13.

*3-(4-Nitrophenyl)-8-propylpyrimido[4,5-c]pyridazine-5,7(6H,8H)-dione* (**8c**): Yield: 54%; m.p. 190–192 °C; IR (KBr) ν_max_ (cm^−1^): 3176 (NH), 3063 (CH arom), 2965, 2873 (CH aliph), 1684, 1595 (C=O), 1449 (C=C), 1510, 1332 (NO_2_), 851 (*p*-substituted); ^1^H-NMR (DMSO-*d*_6_) δ ppm: 12.08 (s, 1H, NH), 8.69 (s, 1H, arom), 8.52 (d, 2H, *J* = 8.8 Hz, arom), 8.38 (d, 2H, *J* = 8.8 Hz, arom), 4.31 (t, 2H, *J* = 7.4 Hz,NCH_2_), 1.77–1.74 (m, 2H, *J* = 7.4 Hz, CH_2_), 0.97 (t, 3H, *J* = 7.4 Hz, CH_3_); MS: *m*/*z* (%) = M^+^, 327 (13), 286 (44), 285 (100), 243 (14), 242 (90), 40 (24); Anal. Calcd for C_15_H_13_N_5_O_4_: C, 55.05; H, 4.00; N, 21.40. Found: C, 55.12; H, 4.09; N, 21.57.

### 3.2. Biological Evaluation

#### 3.2.1. Antimicrobial Bioassay by Using the Agar Diffusion Cylinder Method [36]

All microbial strains were provided from the culture collection of the Regional Center for Mycology and Biotechnology (RCMB), Al-Azhar University, Cairo, Egypt.

The newly-synthesized target compounds were tested in vitro against different types of bacteria, *Streptococcus pneumoniae* and *Bacillus subtilis* as examples of Gram-positive bacteria, and *Pseudomonas aeruginosa* and *Escherichia coli* as examples of Gram-negative bacteria. Fungi, as well as bacteria, were used for testing the antifungal activity of the synthesized compounds. *Aspergillus fumigates* and *Candida albicans* were used as example of fungi and yeast, respectively. The stock solution of concentrations (1 mg/mL) of the synthesized compounds were used. The plates were incubated at 37°C for 24 h for bacteria and yeast, and for 48–72 h for fungi. Tetracycline was used as the standard antibacterial drug while amphotericin B was used as the standard antifungal drug. The diameters of the inhibition zones (mm) were measured and used as criterion for the antimicrobial activity.

#### 3.2.2. Determination of the Minimum Inhibitory Concentration (MIC)

Serial dilutions of the promising compounds were subjected to MIC determination. The different concentrations of each compound were tested with the modified agar diffusion cylinder method as was described before.

#### 3.2.3. Evaluation of the Antitumor Activity Using Viability Assay

All human anticancer cell lines were obtained from the American Type Culture Collection. The cells were grown on RPMI-1640 medium supplemented with 10% inactivated fetal calf serum and 50 µg/mL gentamycin. The cells were maintained at 37 °C in a humidified atmosphere with 5% CO_2_ and were subcultured two to three times a week. For antitumor assays, the tumor cell lines were suspended in medium at concentrations of 5 × 10^4^ cell/well in Corning^®^ 96-well tissue culture plates, then incubated for 24 h. The tested compounds were then added into 96-well plates (three replicates) to achieve eight concentrations for each compound. Six vehicle controls with media or 0.5% DMSO were run for each 96-well plate as a control. After incubating for 24 h, the numbers of viable cells were determined by staining the cells with crystal violet [37,38], followed by cell lysing using 33% glacial acetic acid and read the absorbance at 590 nm using microplate reader (Sunrise, TECAN, Inc., Morrisville, NC, USA) after well mixing. The percentage of viability was calculated as [1−(ODt/ODc)] ×100%, where ODt is the mean optical density of wells treated with the tested sample and ODc is the mean optical density of untreated cells. The relation between surviving cells and drug concentration is plotted to obtain the survival curve of each tumor cell line after treatment with the specified compound. The 50% inhibitory concentration (IC_50_), the concentration required to cause toxic effects in 50% of intact cells, was estimated from graphic plots [37].

## 4. Conclusions

The newly synthesized compounds of indeno[2,1-*c*]pyrimido[5,4-*e*]pyridazines, oxoindolinylidene hydrazinyl pyrimidines, pyrazolo[3,4-*d*]pyrimidines and pyrimido[4,5-*c*]pyridazines were prepared by a simple method. The novel compounds were screened for both antimicrobial and anticancer activities. Compound **4b** showed a very high MICs in comparison to the standard drug tetracycline. Compounds **4a**, **4c** and **8a** exhibited potent antitumor activity against breast cancer in comparison to the standard drug 5-flourouracil.

## Figures and Tables

**Table 1:** In vitro antimicrobial activity of the tested compounds by well diffusion agar assay expressed as inhibition zone diameter (mm) in the form of mean ± SD *.

Tested Compounds	Gram-Positive Bacteria		Gram-Negative Bacteria		Fungi	
	*Bacillus subtilis*	*Streptococcus pneumoniae*	*Escherichia coli*	*Pseudomonas aeruginosa*	*Candida albicans*	*Aspergillus fumigatus*
**4a**	20.6 ± 0.63	18.3 ± 0.72	20.6 ± 1.2	15.2 ± 0.58	NA	18.3 ± 1.5
**4b**	25.3 ± 1.2	22.6 ± 0.72	28.3 ± 0.72	24.2 ± 0.58	NA	23.4 ± 1.5
**4c**	23.6 ± 0.63	21.1 ± 1.5	23.4 ± 1.2	19.2 ± 1.5	NA	21.5 ± 1.2
**5a**	21.3 ± 0.72	22.4 ± 0.63	21.3 ± 0.37	20.1 ± 0.63	NA	19.3 ± 0.63
**5b**	18.1 ± 0.63	17.3 ± 0.63	NA	17.3 ± 0.63	NA	NA
**6b**	22.3 ± 1.5	20.1 ± 0.58	22.4 ± 0.58	18.6 ± 1.2	NA	21.3 ± 1.2
**6c**	17.3 ± 0.63	19.2 ± 0.72	16.3 ± 0.46	17.3 ± 0.63	NA	17.3 ± 0.63
**6d**	20.9 ± 1.5	19.2 ± 1.2	21.3 ± 0.37	17.3 ± 0.63	NA	18.9 ± 1.2
**6f**	15.2 ± 0.63	NA	NA	NA	NA	13.6 ± 0.63
**7a**	NA	NA	NA	NA	NA	NA
**7b**	NA	NA	NA	NA	NA	NA
Tetracycline	28.7 ± 0.5	26.4 ± 0.7	30.2 ± 0.6	27.4 ± 0.8		
Amphotericin B	-	-	-	-	25.4 ± 0.63	23.7 ± 0.72

* NA: No activity under the screening conditions; -: Not tested.

**Table 2:** The MIC of the synthesized compounds.

Sample Tested Microorganisms	4a	4b	4c	5a	6d	Standard
	Minimum Inhibitory Concentration (µg/mL)					
Fungi						Amphotericin B
*Aspergillus fumigatus* (RCMB 02568)	7.81	1.95	3.9	3.9	3.9	1.95
*Candida albicans* (RCMB 05036)	NA	NA	NA	NA	NA	0.98
Gram Positive Bacteria:						*Ampicillin*
*Streptococcuspneumonia* (RCMB 010010)	7.81	1.95	3.9	3.9	3.9	1.95
*Bacillis subtilis* (RCMB 010067)	3.9	0.98	0.98	1.95	3.9	0.49
Gram negative Bacteria:						*Gentamicin*
*Pseudomonas aeruginosa* (RCMB 010043-5)	62.5	0.98	3.9	3.9	15.63	0.98
*Escherichia coli* (RCMB 010052-6)	3.9	0.49	1.95	1.95	3.9	0.49

* NA: No activity.

**Table 3:** The in vitro inhibitory activity of tested compounds against breast carcinoma cell line (MCF-7) expressed as IC_50_ values (μg/mL) ± standard deviation from three replicates.

Tested Compounds	IC_50_ Values (μg/mL)	±Standard Deviation
**4a**	3.6	0.4
**4b**	47.6	2.8
**4c**	4.6	0.3
**5a**	>200	>8
**5b**	95.1	2.6
**6a**	106.7	2.5
**6b**	42.2	1.9
**6c**	160.4	5.8
**6d**	189.9	7.6
**6e**	49.8	2.4
**6f**	34.8	3.2
**7a**	104.5	4.9
**7b**	68.1	1.7
**8a**	20.4	0.8
**8c**	86.1	1.7
5-Flurouracil	4.1	0.6
